# Supplementary material for: Revisiting the Radiosynthesis of [18F]FPEB and Preliminary PET Imaging in a Mouse Model of Alzheimer’s Disease
Source: Molecules. 2020 Feb 22;25(4):982. doi: 10.3390/molecules25040982 (PMC7070414; doi:10.3390/molecules25040982)
Supplement: Supplementary file 1 [file molecules-25-00982-s001.pdf]

molecules

Supplementary information

Revisiting the Radiosynthesis of [^{18}F]FPEB and Preliminary PET Imaging in a Mouse Model of Alzheimer's Disease

Cassis Varlow [1,2], Emily Murrell [1], Jason P. Holland [3,4], Alina Kassenbrock [3],Whitney Shannon [1,5], Steven H. Liang [3], Neil Vasdev [1,3,6,*] and Nickeisha A. Stephenson [1,3,7,*]

[1] Azrieli Centre for Neuro-Radiochemistry, Brain Health Imaging Centre, Centre for Addiction and Mental Health, Toronto, M5T 1R8 ON, Canada; cassis.varlow@mail.utoronto.ca (C.V.); emily.murrell@camhpet.ca (E.M.); wes728@mail.usask.ca (W.S.)

[2] Institute of Medical Science, University of Toronto, Toronto, M5S1A8 ON, Canada

[3] Division of Nuclear Medicine and Molecular Imaging, Massachusetts General Hospital & Department of Radiology, Harvard Medical School, MA 02114 Boston, MA, USA. jason.holland@chem.uzh.ch (J.P.H.); alinakassenbrock@gmail.com (A. K.); Liang.Steven@mgh.harvard.edu (S.V.L.)

[4] Department of Chemistry, University of Zurich, 8057 Zurich, Switzerland

[5] Department of Chemistry, University of Saskatchewan, Saskatoon S7N OX2, SK, Canada

[6] Department of Psychiatry, University of Toronto, Toronto M5T-1R8, ON, Canada

[7] Department of Chemistry, The University of West Indies at Mona, Kingston, Jamaica

*Correspondence: neil.vasdev@utoronto.ca (N.V.); nickeisha.stephenson@uwimona.edu.jm (N.A.S.); Tel.: 416-535-8501 ext. 30988 (N.V.); 1-876-927-1910 (N.A.S)

FXN HPLC Traces

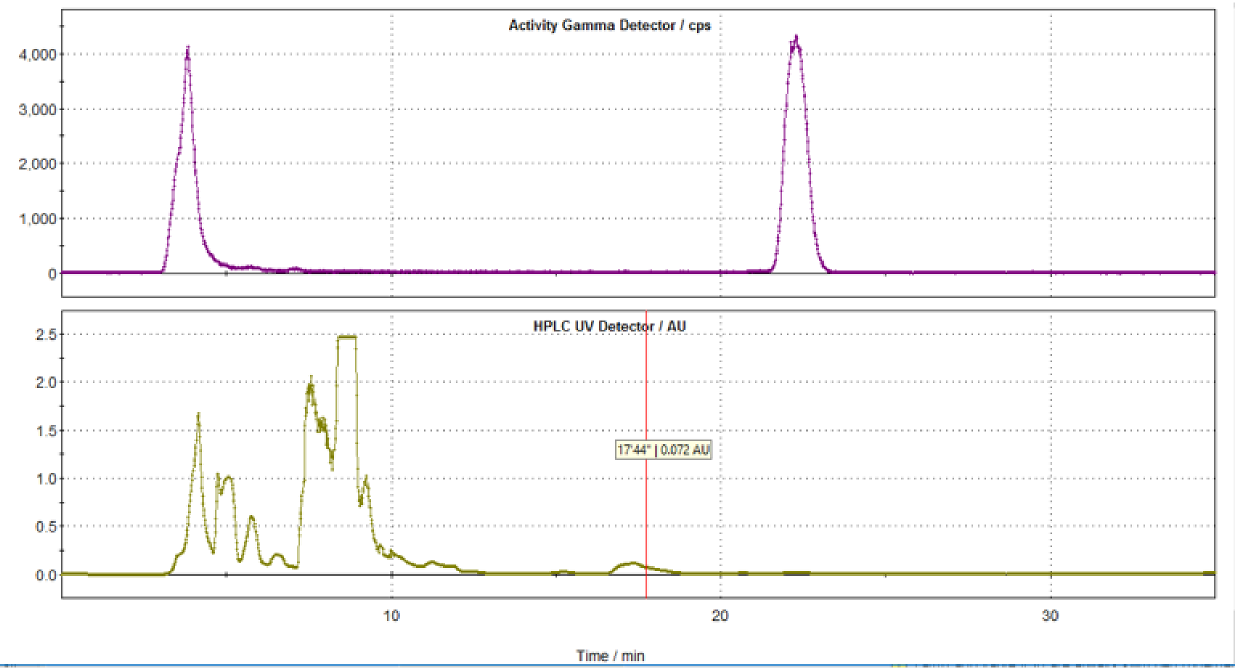

Figure S1. Semipreparative HPLC trace of a typical radiosynthesis of [^{18}F]FPEB using precursor (**5**).

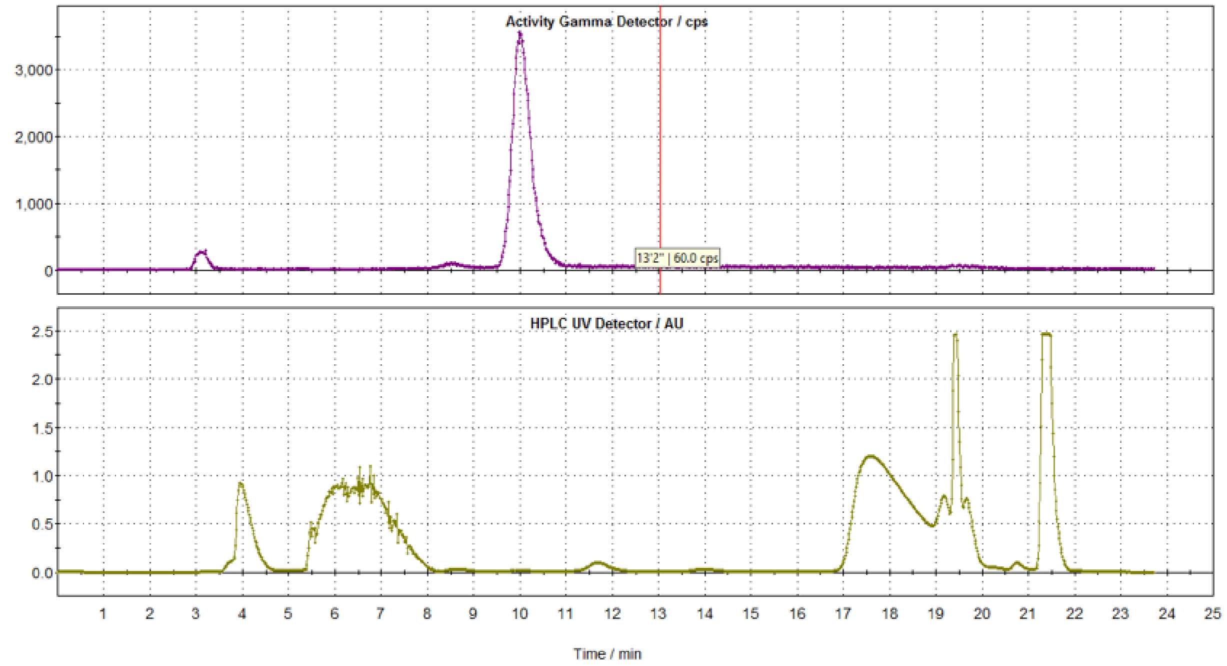

Figure S2. Semipreparative HPLC trace of a typical radiosynthesis of [^{18}F]FPEB using precursor (**6**).

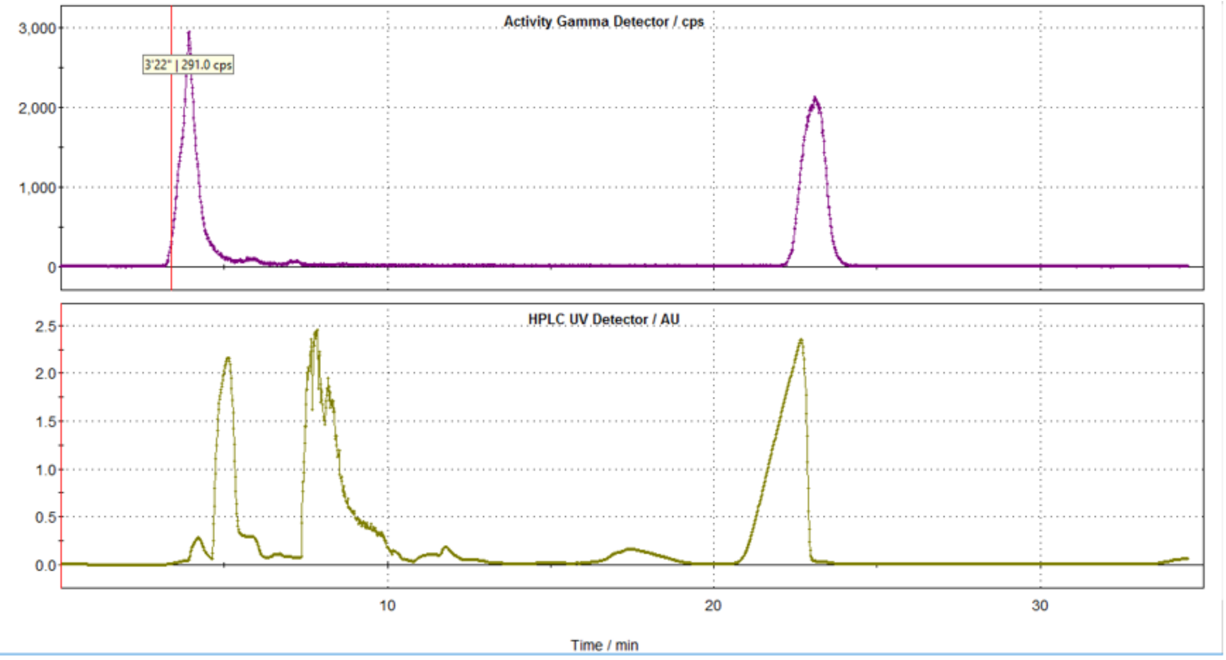

Figure S3. Semipreparative HPLC trace of a typical radiosynthesis of [^{18}F]FPEB using precursor (**7**).
